# Self-Centered Function of Adaptive Immunity in Regulation of Immune Responses and in Tolerance

**DOI:** 10.1155/2021/7507459

**Published:** 2021-12-02

**Authors:** Silvana Balzar

**Affiliations:** Department of Clinical Microbiology, Polyclinic Breyer, Zagreb, Croatia

## Abstract

The search for common mechanisms underlying the pathogenesis of chronic inflammatory conditions has crystalized the concept of continuous dual resetting of the immune repertoire (CDR) as a basic principle of the immune system function. Consequently, outlined was the first dynamic comprehensive picture of the immune system function. The goal of this study is to elaborate on regulation of immune responses and mechanisms of tolerance, particularly focusing on adaptive immunity. It is well established that the T/B cell repertoire is selected and maintained based on interactions with self. However, their activation also requires interaction with a self-specific major histocompatibility complex (MHC) “code,” i.e., the context of MHC molecules. Therefore, not only repertoire selection and maintenance but also the T/B cell activation and function are self-centered. Thus, adaptive effectors may be primarily focused on the state of self and maintenance of integrity of the self, and only to a certain degree on elimination of the foreign. As examples of such function are used immunologically poorly understood MHC-disparate settings typical for transplantation and pregnancy. Transplantation represents an extreme setting of strong systemic compartment-level adaptive/MHC-restricted immune responses. Described are clinically identified conditions for operational tolerance of MHC-disparate tissues/living systems in allotransplantation, which are in line with the CDR-proposed self-centered regulatory role of T/B cells. In contrast, normal pregnancy is coexistence of semiallogeneic or entirely allogeneic mother and fetus, but without alloreactivity akin to transplantation settings. Presented data support the notion that maintenance of pregnancy is a process that relies predominantly on innate/MHC-independent immune mechanisms. By the inception of hemotrophic stage of pregnancy (second and third trimester), both mother and child are individual living systems, with established adaptive immune repertoires. Although mother-fetus interactions at that point become indirect systemic compartment-level communications, their interactions throughout gestation remain within the innate realm of molecular-level adaptations.

## 1. Introduction

The concept of continuous dual resetting of the immune repertoire (CDR) as a basic principle of the immune system function outlines a comprehensive, dynamic picture of the immune system function that is governed by the randomness of interactions and uncertainty of outcomes [1]. The original paper focuses on applying CDR to describe common mechanisms underlying the pathogenesis of chronic inflammatory conditions and autoimmune diseases, including processes associated with both pathologic and aging-related immunosenescence. It also defines the elusive immunological self and describes the dynamics of regulation of immune responses and tolerance [2]. This paper's intention is to further develop the notion that adaptive effectors represent a high-level regulatory mechanism to maintain integrity of a living system. Discussed will be the role of major histocompatibility complex (MHC) in the proposed primary focus of adaptive immunity on the state of self. Prominent examples of regulation of immune responses and maintenance of states of tolerance/integrity of a living system(s) will be used allogeneic settings inherent to both, transplantation and pregnancy. First segment of this paper will tackle the coexistence of MHC-disparate tissues in transplantation and then proceed to analyze the physiologic setting that supports semiallogeneic or entirely allogeneic pregnancy.

This paper briefly outlines the segments of the CDR pertinent to discussed issues. However, broader familiarity with the CDR is advisable.

### 1.1. Getting Priorities Straight: Maintenance of Integrity Supersedes Elimination of the Foreign or Dangerous

The CDR puts immunity into a more general context of maintenance of organism's integrity instead of perceiving immune processes as a battle against the foreign or dangerous. It outlines a rather pacifistic picture of the immune system function: continuous molecular-level resetting/adjustments in response to molecular-level changes/disturbances instead of antagonizing them. Disturbances that may trigger the immune repertoire resetting include (a) interactions with the environment and (b) intrinsic changes of self. There is no inherent animosity against intruders or unknowns. Although perceived as such, the drive to destroy or kill often used to describe immune reactions is not congruent with nature's intrinsic mechanisms of adaptation. Instead, the main purpose of immune responses is about overcoming a disturbance (regardless of its nature) with minimum energy expenditure, and guiding innate responses (sometimes through actions of adaptive immunity) toward equilibrium/steady-states.

Such a seemingly subtle shift in understanding immunity has complex implications. This CDR-driven concept of integrity maintenance versus elimination/neutralization of everything sensed as a foreign is with an understanding that disturbances are not just the foreign. Disturbance can be also physiologic growth, hormonal effects, pregnancy, mechanical injury, etc. The system continues resetting toward more energy-efficient states, which are never the same as before. The change is continuous and a constant in living system's existence, and there is no going back to previous states. In response to disturbances, the system takes thermodynamically optimal path to acquire appropriate steady-states. Those steady-states may not be always perceived as states of health but are the optimum for given parameters and under given circumstances.

## 2. Transplantation

Despite significant advances in transplantation approaches, conditioning procedures that deplete immune system of recipients, continuous immunosuppression required in many patients, and paucity of reliable markers to guide clinicians in decisions about caring for their patients make transplantation a difficult process with uncertain outcomes. Operational tolerance that results in a stable long-term function without the need for immunosuppression remains difficult to achieve. Recent clinical studies demonstrate that induction of a stable mixed chimerism or inclusion of donor's liver in the combined transplantation with other parenchymal organs improve transplantation outcomes [3–9]. However, underlying immunological processes associated with tolerance remain unclear. The most limiting issue is the lack of a general understanding of immunity, which would provide more grounded rationale for various transplantation approaches.

### 2.1. Conventional Adaptive Immunity in Maintenance of Integrity: the Importance of Knowing Thy Self

The CDR describes regulation of adaptive responses through fluctuations in the phenotype profile of a T cell receptor- (TCR) diverse T cell population activated in a particular adaptive response, so that immune response eventually enters the phase of repair and resolution [1]. How does understanding of the T cell function as focused on the maintenance of system's integrity makes a difference as compared to the perception of T cells as focused on a particular antigen and its elimination?

**Figure 1:**
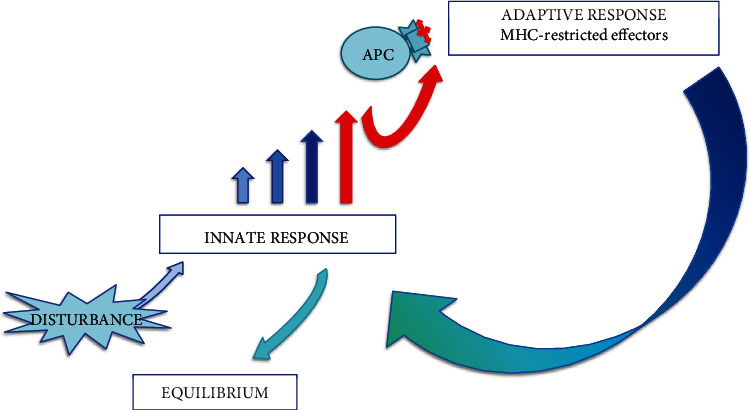
Activation of the innate-adaptive-innate regulatory loop. Innate signaling above the threshold activates adaptive responses (conventional T/B lymphocytes) to regulate innate responses toward resolution and new equilibrium states.

It is well established that T cell repertoire is selected based on interactions with self and therefore mirrors the self. This “self-obsession” continues as a requirement for homeostatic signaling from interactions with self (self-awareness), which is necessary in maintenance of T cell repertoire/specificities in the periphery [10]. Surprisingly, implementation of the CDR leads to a conclusion that not only T cell repertoire selection and its maintenance are self-centered, but the T cell function is self-centered as well: T cell repertoire, knowing/mirroring the self and responding only to MHC-restricted innate alerts, may primarily focus on the state of self (hence the MHC restriction of a self-based adaptive repertoire that is useless in a MHC-mismatched host). Here, it is important to keep in mind that the T cell repertoire selection in thymus proceeds through interactions between the MHC-bound self-antigens presented by thymic epithelial cells and the randomly assembled TCRs on newly formed T cells. The MHC is always a component of the molecular pattern that interacts with TCRs, i.e., TCRs recognize epitopes only in the MHC context. Therefore, MHC serves as a “code” that allows cognate interactions with T cells, but only with the code-matching antigen-presenting cells (APCs). The MHC code is unique to a particular living system, which implies that information exchanged through MHC-restricted communications is pertinent only to that particular living system and directed toward regulation of its integrity. Thus, such communication regulates innate immune responses in the context of self and toward maintenance of self's integrity. More precisely, as APC-delivered antigen presentation does not discriminate self/altered self from the foreign, activation of T cell specificities in response to foreign epitopes is due to cross-reactivity of the T cells' self-mirroring repertoire with the foreign, as well as with the APC-presented damaged self. Thus, T cell activity is determined by the self and proceeds in the context of self regardless whether elicited by a foreign or by the self-antigens. Innate mechanisms/effectors, guided and regulated by T/B cells, are actually doing the basic work—eliminating infectious agents, removing destructed tissue, etc. The self-regulating loop closes when innate signals, modulated by T/B cells' regulatory capacity (determined by their repertoire's granularity), eventually change the quality/intensity of integrated signaling toward T/B cells and terminate their engagement. So, it is the innate→adaptive→innate mutuality of signaling/interactions that eventually establish homeostasis (Figure 1).

How this view of immunity applies to transplantation? In a recipient, the presence of allotransplant is first sensed by innate mechanisms as a major disturbance in a system. It is not only the foreign but also the trauma caused by the procedure that could overwhelm the system and take it toward the loss of integrity/death. As immune reactions are triggered by mechanisms that do not distinguish between disturbances caused by the foreign or the damaged self, minimizing the injury during initial stages of transplantation (recipient's trauma by the diseased organ resection and graft attachment; graft's trauma by detachment and subsequent reperfusion injury) may be beneficial. Establishing an acceptable pace of innate reactions could allow appropriate time for resetting/adaptation to new circumstances on both recipient's and graft's sides. As regulatory mechanisms of adaptive responses are to engage later in the process, important element in successful regulation of innate responses would be a diverse repertoire of adaptive effectors on both, recipient's and donor's sides. The dynamics and outcomes of those processes are uncertain. However, some level of control of the pace of those initial innate and consequent adaptive processes may help the system to overcome such a major disturbance and gradually establish equilibrium states compatible with integrity of the newly created chimeric system.

As CDR posits, T/B cells' function is primarily to guide innate immune responses toward homeostasis, resolution, and repair. Their self-centered repertoire determines the system's ability to maintain integrity, i.e., through continuous resetting maintain the states of homeostasis. Being evolutionary developed only in most complex and highly organized living systems (yawed vertebrates) further supports the notion that the range of T cell phenotypes may represent a self-centered regulatory mechanism to maintain integrity of those most complex living megaorganisms. This view of T/B cell function may explain consequences of iatrogenic or disease-induced lymphopenia. Lymphopenic patients become susceptible and often succumb to infections with microorganisms considered commensals or environmental flora (*Candida* species, *Pseudomonas aeruginosa*, and *Pneumocystis jirovecii*), possibly due to unregulated innate responses. Depleted T cell population, and thus reduced repertoire, may result in inability to regulate/channel innate responses toward maintenance of system's integrity. Similar effects of lymphopenia in a transplant recipient could jeopardize regulatory mechanisms required to establish equilibrium states and eventual graft tolerance. In addition, transplant's integrity (as explained below) may be similarly affected.

### 2.2. Maintenance of Integrity in Transplantation

How the proposed function of self-centered adaptive repertoire in maintenance of system's integrity can be applied to understand issues in transplantation? In a transplanted organ that lacks autochthon T cells and their MHC-matched APCs (T cells + MHC − matched APCs = TAPCs), transplant's disturbances (ischemic injury, innate alloreactions, etc.) remain unattended/unregulated, because the transplant is outside the “jurisdiction” of host's TAPCs. Transplant remains a stranger to host's TAPCs. Even when relative homeostasis can be established, specific regulatory mechanisms to preserve integrity of a transplant (would be in the domain of its own TAPCs) may not function appropriately, and integrity of a transplant may be brittle. Although survival can be prolonged, it would be in the context of host's integrity.

If the graft/transplant brings its own self-focused/integrity-preserving TAPCs into a host with functioning TAPCs (that regulate host's innate responses and watch over host's integrity), the resetting processes may be directed toward steady-states that would allow coexistence of both entities—the host and the transplanted organ. Such a setting may exist in liver transplantation. Liver, due to its complex function as an immunological barrier between the gut's mucosal compartment and the systemic compartment, has its own resident population of lymphocytes and APCs. That puts liver in a position to regulate its own resetting processes toward maintaining its own integrity versus host's own immune mechanisms maintaining host's integrity. Although (allo)reactions (including host vs. graft and graft vs. host) may continue, regulatory mechanisms on both sides may eventually reach sustainable steady-states and allow both MHC-distinct entities to coexist. Liver's TAPCs, supported by their autochthon environment, are likely to continue their function even in a MHC-mismatched host. Spontaneous operational tolerance of liver transplants is indeed more common than with other parenchymal organ transplants [9].

The proposed benefit of having functional both host and donor TAPCs is demonstrated in a particular approach to transplantation of organs that, unlike liver, lack autochthon TAPCs. It has been recognized that establishing stable mixed chimerism in organ transplantation may reduce complications and help achieve tolerance [3–7]. Reports suggest that establishing stable mixed chimerism with infusion of donor haematopoietic stem cells (HSC)+T cells in a solid organ recipient can prevent rejection, eventually lead to cessation of immunosuppressive therapy and induction of transplant tolerance [3–5]. Preserved recipient's T cell repertoire, B cell lymphopoiesis, and myelopoiesis may be the source of recipient's replenished population of lymphocytes and APCs undergoing posttransplant resetting against the donor's molecular patterns. In the case of B cells, the resetting process may include de novo posttransplant repertoire selection/replenishment. The observed strong B cell signature in subjects with operationally tolerant kidney transplants originates from naïve and transitional B cells, which suggests that indeed newly derived B cell repertoire may be resetting against the newly present alloentity [11, 12]. At the same time, infused donor's HSC+T cells may be resetting against the recipient's environment, looking for the way to engraft and find a “home” for donor's TAPCs. Successful resetting processes may result in mixed chimerism, with both recipient's and donor's TAPCs providing maintenance of respective immunologically disparate entities that coexist within the recipient's body. The innate (allo)reactions on both sides would be regulated by the respective MHC-matching TAPCs, while functioning in the context of preserving integrity of the newly created chimeric megaorganism. While outcomes are uncertain, the CDR-postulated intrinsic lack of animosity toward the foreign and intrinsically regulated resetting processes toward energy-efficient steady-states may result in persistence/maintenance of integrity of a chimeric megaorganism. Indeed, contribution of both recipient and donor cells to the population of peripheral blood regulatory T cells (Tregs) has been recently reported in subjects with mixed chimerism post HSC transplantation [13].

Consistent with the CDR's understanding of graft tolerance and importance of having both donor and recipient MHC-matched TAPCs to maintain the integrity of a chimeric living system, the full donor chimerism associates with graft-versus-host disease, recipient's immunodeficiency and immune incompetence, suggesting that donor's leucocytes may not be appropriate/sufficient to maintain recipient's integrity [6, 7, 14].

It has been observed that tolerance can be lost after years of stable allograft function. In some subjects, the triggers were viral or bacterial infections, while others can develop immunologically driven rejection [15, 16]. Indeed, conclusions drawn from transplantation studies indicate that tolerance is an acquired and metastable condition [17]. These clinical observations are consistent with the CDR view of tolerance as a dynamic process maintained by a continuous resetting of the immune repertoire in a system whose function is governed by randomness of events and uncertainty of outcomes. Significant disturbances in the system (such as infection or even allograft biopsy procedure) may carry a risk of resetting processes that could trigger rejection. The rejection could be due to reduced regulation of reactions unrelated to the transplant itself (bystander effects due to cross-reactivity triggered by infection, transplant-unrelated injury), and also due to physiologic reduction in repertoire diversity related to aging [1]. Consistent with uncertainty of living system's function, those events could occur at any point in time.

As mentioned, equilibrium states are established by thermodynamically optimal mechanisms, which could include markers otherwise associated with pathology. Nevertheless, those may reflect equilibrium states still compatible with maintenance of integrity. In that context, biopsy-detectable indicators of continuing alloreactivity despite clinically evident operational tolerance may not be surprising or a sign of rejection. Therefore, routine biopsy to assess the state of a transplant may not be as useful/informative, particularly considering the risk that may carry. Perhaps looking for peripheral blood markers of innate activation reflective of the transplant-related resetting processes (and indicators of potentially significant disturbance leading to activation of adaptive reactions) may constitute a better warning system of possible rejection. As each patient's immune states are expected to be unique, personalized pre- and posttransplant “baselines” for future follow-up may need to be established. For that purpose, identifying the parameters most indicative of pretransplant immunological setting/background (may be influenced by gender, age, genetics etc.; ref. 1) and the posttransplant states may require multifactorial profiling/analyses of many patients.

While the “self-obsession” and self-awareness hint at basic requirements, what exactly constitutes the homeostatic signaling that gives a sense of home for lymphocyte/APC populations (and thus may create a refuge for donor cells that results in engraftment) is unknown [10]. As liver can be a home and a source of donor's TAPCs, perhaps an organ transplant with adjacent lymph nodes or lymphoid tissue could provide similar homing sites and a source of transplant-preserving donor TAPCs for maintenance of a stable mixed chimerism.

### 2.3. Concluding Remarks regarding Transplantation

Under appropriate conditions, immune interactions may result in acceptance of an allotransplant and persistence/maintenance of integrity of a chimeric organism. Having in mind that stochasticity and chaotic behavior govern the function of living systems, iatrogenic elimination of particular segments of those processes may not be a good strategy to achieve better outcomes. Instead, supporting the living system's resetting mechanisms and directing them toward outcomes that would be less detrimental for system's integrity may be a more productive strategy. These issues may be of particular interest for approaches to modulate early posttransplant resetting processes. The goal would be to skew those initial “raw state” processes toward equilibrium states conducive to better clinical outcomes instead of allowing the system to take a direct “shortcut” toward acute situation-appropriate equilibrium states that may associate with unacceptable pathology, rejection, or loss of integrity/death [18].

The proposed self-centered function of conventional T/B cells represents an upper-tier regulatory mechanism that is engaged only under circumstances that require channeling innate responses toward maintenance of living system's integrity. Therefore, a diverse repertoire of T/B cells and MHC-matching APCs to regulate interactions and resetting processes of both entities in the newly created chimeric living system may be essential. That means that preserving adaptive repertoire of both the recipient and the donor may be necessary to acquire an immunosuppression-free operational tolerance. In that context, donor-specific antibodies may be a part of those regulatory processes and not necessarily an indicator of rejection. Indeed, while donor-specific anti-human leukocyte antigen (HLA) antibodies may associate with graft loss, they do not reliably predict allograft rejection [17]. Unlike with T cells (whose repertoire is limited by thymus involution), the B cell repertoire continues to be replenished throughout one's life, which makes them a more “adaptable” element of regulatory processes and tolerance. Also, antibodies, regardless of origin, may bind both donor's and recipient's epitopes and thus contribute to the regulation of immune responses.

Considering that the T cell repertoire is formed at the end of individual's growth/maturation (which also coincides with involution of thymus), transplant tolerance in humans is operational tolerance. It depends on repertoire's granularity/diversity [1]. The role of the thymus and central tolerance in humans could be potentially a factor only in very young children, while the thymus is still active. Proposals to reactivate the function of thymus in adults (and thus derive/select new Tregs) are disconcerting, as clinical data show that activation of thymus in adults associates with autoimmunity [19–21].

Association between the increase in Tregs and transplant tolerance has resulted in approaches to condition polyclonal T cells *in vitro* to acquire a regulatory phenotype and use those Tregs to induce transplant tolerance *in vivo*. Considering that the phenotype of all cells, including T cells, forms and fluctuates as a result of integrated signaling received from cells' environment, potential regulatory effect of *in vitro*-conditioned Tregs may not translate to *in vivo* setting.

## 3. Pregnancy

Immunologically, pregnancy is a puzzling physiologic phenomenon. Contrary to incompatibility between allogeneic tissues that makes transplantation such a challenging and uncertain process, a woman's body cradles and supports the growth of not only her own semiallogeneic fetus but, in gestational surrogacy, supports an allogeneic fetus created from other woman's oocyte. In transplantation, an allogeneic transplant can only rarely maintain its function without immunosuppression. Pregnancy, a basic physiologic event, readily supports the development of an MHC-disparate fetus. Mechanisms involved in that physiologic process are still poorly understood.

Per CDR, rather than acting against unknowns, immunity includes a molecular-level resetting process of adaptation to disturbances in the system. Maintenance of integrity (i.e., immunity) is a matter of adaptation to changes. Also, regardless whether (innately) detected changes are due to continuous changes of self or originating from the environment (including infectious agents and allogeneic interactions), there is no intrinsic animosity: reactions are self-regulated, and their outcomes depend on the living system's immune repertoire and its competency. Such a pacifistic understanding of immunity, as opposed to the presumption of intrinsic aggression against unknowns, allows for a different view of pregnancy, as well as transplant tolerance. Therefore, per CDR, pregnancy is a matter of adaptation to an allogeneic entity—conceptus/fetus.

### 3.1. Compartmentalization of the Immune System: the Importance of Sequestration of the Systemic Compartment

An important element of the CDR to emphasize before discussing the immunological aspects of pregnancy is compartmentalization of the immune system. Compartmentalization is essential in maintenance of living system's integrity [1]. The surface-lining mucosal compartment (MC) functions as a barrier against the environment and keeps the systemic compartment (SC) sequestered/isolated. Equipped with a particular population of unconventional cells and innate mechanisms to interact with the environment, MC could be considered an immune-privileged site where more is “allowed” without triggering an adaptive response/engagement of the systemic effectors.

**Figure 2:**
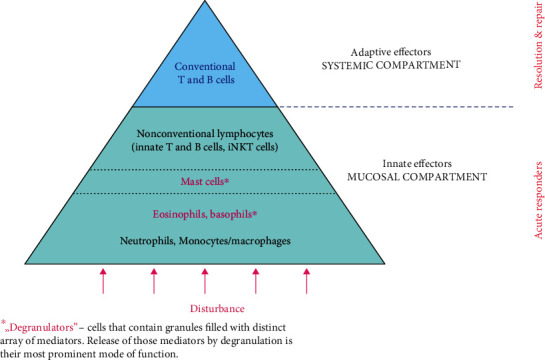
The engagement sequence pyramid of cellular immune effectors. Physiologic characteristics of immune cells, their timing of recruitment, number, and distribution are consistent with their function. Innate effectors are the first/acute responders. Conventional T/B cells (MHC-restricted adaptive effectors) are secluded within the systemic immune compartment (SC) and activated only when innate reactions reach a particular threshold, i.e., in later stages of an immune response.

Figure 2 outlines the hierarchy in activation of effector mechanisms (in the domain of cellular immune responses) during the course of an immune response, which is clinically evident in typical dynamics of an acute inflammatory reaction. As a rule, and regardless whether the trigger is infection or a noninfectious injury/disturbance, early stages of an acute inflammatory reaction are marked by a prompt increase in neutrophils/granulocytes (left shift). Neutrophil/granulocyte recruitment and influx are an innate prompt reaction to a range of infectious or noninfectious disturbances in the system. While relatively short-lived, their role has been proven indispensable [22]. Although not as numerous as neutrophils, eosinophils and basophils (highlighted as “degranulators”) emerge as unique innate cells that are engaged in immunologically challenging reactions triggered at mucosal surfaces (allergy, responses to parasites), and also in regulation of repair and profibrotic processes [23]. Mast cells, unlike granulocytes, constitute a resident population of cells distributed along epithelial and endothelial surfaces. They are considered essential for homeostasis and barrier function of the mucosal immune compartment (MC) [24–26]. Nonconventional T/B cells represent an innate segment of local regulatory mechanisms that function within the MC. Unlike conventional T/B cells, they are directly activated by innate signals/interactions and are able to act promptly.

Eventual lymphocytosis (when adaptive arm of immunity is engaged) is established several days later and is considered a marker of late stages of an inflammatory reaction. Activation of conventional T cells is MHC-restricted. The highly regulated activation and clonal expansion make the conventional lymphocytes slow-reacting effectors reserved for processes that cannot be resolved solely by innate mechanisms, i.e., those innate processes that reach a threshold of signaling indicating significant disturbance in the system (Figure 1). Conventional T cells require interactions with APCs-activated cells capable to pre-process antigens and present those in the context of MHC molecules. Similarly restricted conventional B cells require cognate T cell help to undergo class switch and specificity maturation (somatic hypermutation).

Clinically, a drop in peripheral blood neutrophils and concomitant lymphocytosis associate with resolution and recovery. In infectious diseases, the drop in peripheral blood neutrophils coincides also with the specific disease-associated seroconversion and increase in immunoglobulin G levels. That typically occurs 7-10 days after initial symptoms of the disease. The timing of those routinely measurable effects is consistent with the CDR view: initial strong innate reaction is subsequently regulated by T/B lymphocytes.

The sequestered SC is a home of adaptive T/B cell effectors. Their MHC-guided/focused responses are activated only when disturbances reach a threshold requiring a specificity-driven adaptive reaction (Figure 1). Those adaptive responses, through innate effectors, neutralize and repair the damage, thus regulating inflammatory reactions. Reliance on mucosal/innate protection, with seldom activation of the systemic compartment effectors/adaptive responses, reduces immune repertoire attrition (intrinsic to the CDR) and preserves regulatory capacity of the system. Unlike with transplantation, where recipient-donor interactions occur as a direct systemic event (surgery or parenteral introduction of heterologous cells is a harsh disturbance of the SC), appropriate compartmentalization of the immune system and strong MC may play a major role in implantation and maintenance of pregnancy.

Another important element in discussing the immunologic aspects of pregnancy is the role of glycosylation and glycation, in which CDR considers the driving force in growth, differentiation, immune repertoire development, and aging [1]. Here, the role of glycosylation will be considered related to appropriate decidualization and placentation processes, creation of an immunologically inert uterine environment, and its systemic impact on mother's immune status.

Finally, this study will address how fetal and maternal immune systems' interactions may shape the development of fetal immunity and affect mother's immune status.

### 3.2. First Trimester of Pregnancy—a Mucosal Event

It has been established that the first 10-12 weeks of pregnancy represent a histiotrophic phase, during which the carbohydrate moieties/glycoprotein-rich endometrial/decidual environment are the source of nutrients for the conceptus/fetus [27]. Preimplantation conditioning of endometrium includes a progesterone-driven glycosylation processes, with abundant secretion of highly glycosylated moieties. Histologic studies show uterine epithelium immersed in mucous secretion [27]. Mucous secretion at other mucosal surfaces (respiratory and digestive tract) functions as a protective and antigen-exclusion mechanism. It renders epithelial surfaces less reactive to environmental triggers, which modulates epithelial signaling to stromal cells and thus modulates the mucosal environment. That, in turn, shapes the phenotype and function of resident immune cells and regulates the influx of other cells involved in immune reactions/responses [1]. While other mucosal surfaces are directly exposed to the environment and their mucous secretion is triggered by exogenous stimuli, endometrium is largely protected from such exposures. Endometrial mucous secretion is endogenously (due to hormonal changes) induced to create a pregnancy-conducive environment.

The prominent glycosylation, so obvious in uterine epithelium/glands, may change decidual/stromal environment as well. The process of decidualization creates a particular tissue environment that modifies the phenotype and function of resident cells, including stromal cells, decidual natural killer (NK) cells, and macrophages. The placentation processes under such conditions are governed by appropriate interactions between decidual/stromal environment and both, maternal and fetal cells (trophoblast), and proceed to form an appropriate maternal-fetal interface [28, 29].

Glycosylation, an enzymatic addition of carbohydrate sequences to proteins and lipids, is known to differentially affect all molecular interactions—adhesion, receptor-ligand binding/signaling, innate signaling, etc. [30, 31]. The pregnancy-induced glycosylation may create an immunologically inert environment that is during the first trimester conducive to implantation, propagation of fetal trophoblast deeper into decidual layers, and formation of the placenta. Indeed, recent studies demonstrate differential glycosylation in specific phases of the menstrual cycle, implantation, and in placentation-associated pathology of pregnancy [32, 33]. In addition, reported is a complex role of glycan-binding galectins in placentation and pregnancy disorders [34, 35]. Also, baseline-altered glycosylation and/or glycation (due to aging or other altered glycosylation/glycation states such as diabetes, obesity, and chronic inflammatory conditions) could result in impaired placentation and unfavorable pregnancy outcomes. Indeed, murine reproductive decline in aged dames associates with altered uterine environment, blunted hormonal responsiveness, and thus deficient decidualization and placentation [36]. Those could be due to aging-associated changes in glycation/glycosylation [1].

Therefore, under physiologic conditions, adaptation to the invading alloentity during implantation may proceed regulated solely by innate mechanisms residing within the uterine mucosa. Those innate mechanisms include the molecular-level resetting and the local tissue-modulated interactions between the fetal trophoblast, decidual NK cells, and macrophages. From the CDR perspective, it is important for this mucosal process to remain within the innate realm of interactions and not to engage mother's SC/adaptive immune mechanisms (fetal immunity is at that point still limited to the innate). As emphasized before, adaptive responses are intrinsically autoreactive and thus could not only jeopardize the pregnancy/fetal survival but also hurt the mother.

### 3.3. Second and Third Trimester of Pregnancy: Communication of Maternal and Fetal Systemic Immune Compartments

The blood flow within the newly formed placenta is detectable around 12th week of pregnancy and marks the beginning of the hemotrophic phase of pregnancy [27]. Mother's blood bathes the large surface of intricately branched placenta. The layer of placental syncytiotrophoblast (STB) facing mother's side represents the barrier that from second trimester onward maintains molecular-level communication between the fetus and the mother. In addition to gas, nutrients, and waste exchange, trafficking includes various molecules (environmental particles and other environmental cues), inflammatory mediators, immunoglobulins, exosomes/extracellular vesicles, etc. [37, 38]. Also, a certain number of cells are exchanged between the mother and her developing child [39, 40].

During the histiotrophic phase, mother-fetus interactions are limited: mother's uterine mucosal environment resets against the proliferating and differentiating fetal trophoblast, and vice versa. With the placental blood flow established, the fetal STB begins its adaptation to systemic molecular patterns of mother's self. Also, mother's exposure to the STB is now direct/systemic—not through decidua/MC. In addition to mother-fetus interactions, the STB layer is to modulate interactions with molecular patterns of the environment that may break through mother's mucosal barrier (genital, respiratory, and gut mucosa and skin) and reach the blood circulation. Thus, the strength of mother's MC and its barrier function will directly affect the degree of fetal exposure to the environment. While modulated by STB, both mother's self and the foreign/environment may translate into molecular-level disturbances in the fetus and trigger the resetting processes pertinent to the fetal growth, its immune repertoire development, and maintenance of its integrity.

#### 3.3.1. Fetal Immunity

Fetal immune system's T cell compartment begins to develop at 10 weeks of gestation [41]. That precedes the inception of placental blood flow and increased level of disturbances due to the more direct exposure of STB/fetus to mother's immune environment. The thymus-derived populations of T cells include conventional *αβ* T cells, as well as the innate *γδ* T cells, *αβ* innate natural killer T cells (iNKT cells), and mucosa-associated innate T cells (MAIT cells) [42]. Innate T cells populate fetal mucosal tissues and mature before the postnatal microbial exposures [43]. Similar timing and distribution are observed for myeloid lineages, including innate B1 and marginal zone B cells, monocytes, and macrophages [44].

#### 3.3.2. Mother's Immune Status

A mother's entire body undergoes changes under the influence of pregnancy-induced hormonal environment. The number of various forms of steroid hormones increased in pregnancy (progestogens, estrogens), numerous forms of steroid hormones' receptors, and the well-recognized promiscuity in their interactions makes it difficult to clearly understand the role of hormonal setting typical for pregnancy. That includes the function of the most investigated and clearly essential progesterone [45]. Still, it is reasonable to infer that the progesterone-associated highly glycosylated local/uterine environment may be reflective of a similar pregnancy-induced systemic increase in glycosylation. Systemically increased glycosylation of molecular patterns involved in signaling, matrix composition, and cellular interactions could result in phenomena quite typical for pregnancy. For example, altered function of olfactory and gustatory receptors could result in altered sense of smell or taste in pregnancy; glycosylation-affected insulin receptors could associate with insulin resistance and gestational diabetes; altered epitopes of tissue structural elements could underlie amelioration of rheumatoid arthritis symptoms that is known to occur during pregnancy, etc. Similarly, increased glycosylation may result in generally different tissue/immune environment, which can shift the signaling toward a predominantly Th2 realm of humoral responses, another common feature of pregnancy. Although a Th2 pattern of responses is known to be prominent also in nonpregnant women, in pregnancy, it is considered part of a tolerogenic environment that protects the pregnancy [46]. Indeed, pregnancy may associate with somewhat blunted responses to certain pathogens and increased susceptibility to infections such as listeriosis, malaria, and human immunodeficiency virus (HIV) infections [47]. However, pregnancy is not considered an immunosuppressed state.

### 3.4. Mother-Fetus Interactions: the Balancing Act of Maintaining Integrity

A mother, an immunologically competent living system with a well-developed MC that functions as a strong barrier against the environment, will primarily rely on the innate mechanisms of her MC for appropriate implantation and placentation during the first trimester of pregnancy. The highly glycosylated uterine environment may facilitate the resetting processes of adaptation to the presence of the conceptus. The MC will regulate those innate interactions/disturbances to remain below the threshold of activation of the SC's conventional/adaptive effectors. Fetal trophoblast uses only innate mechanisms of molecular resetting/adaptation to continue growing and invading the decidual layer, the associated remodeling process facilitated by mother's NK cells and macrophages. Such a setting is unlikely to elicit immune interactions that would damage either side.

At the beginning of the second trimester, fetus and mother are individual living systems whose SCs come into close contact—separated only by the fetal STB. Fetus is in the process of developing its own SC's repertoire of T/B cells using its self-template in the selection process in thymus [10]. Its self-template rapidly changes due to endogenous physiologic processes of intense growth and differentiation of fetal organs and tissues. Sequestered/undisturbed from outside, the fetal repertoire selection will produce a self-mirroring repertoire needed for regulation of innate reactions triggered by various disturbances, which will work toward maintenance of fetus' integrity. The STB may information (various mediators, exosomes, immunoglobulins etc.) received from mother's blood “translate” into an instructive element in the process of fetal adaptation to the post-natal life. On the other hand, information from the fetal side toward mother is propagated into a large, quiescent arena of the mother's SC. Unless it contains alarming signals capable of inducing strong innate (and possibly adaptive) reactions, there is little unknown about the fetus that mother would detect as a significant disturbance requiring engagement of her adaptive effectors.

Although mother's strong MC shields the STB and fetus from exposures to the environment, the lack of a more complex/mucosa-like barrier on the fetal side makes the fetus vulnerable. Fetal SC may be exposed to exogenous/environmental disturbances otherwise innocuous for the mother (toxins, chemicals, allergens, and microbial antigens). Those could reach fetal circulation and trigger fetal systemic-level resetting processes. That may alter not only the fetal developmental processes (which, depending on the time of exposure, could cause major structural and functional alterations) but also its self-template and thus the selection of fetal T/B cell repertoire. Outcomes of those fetal systemic-level intrusions could range from the postpartum phenotypically undetectable to significant functional and immunologic alterations in early childhood/adolescence/adulthood. An extreme example of such processes is fetal inflammatory response syndrome (FIRS): mother's mild inflammatory reaction to viral infection (where infection itself does not spread to the placenta/fetus and mother's inflammatory reaction does not terminate the pregnancy) associates with increased risk for diagnosis of autism, schizophrenia, neurosensorial deficits, and psychosis later in life [48]. In that situation, despite the absence of placental transmission of infection, the sole exposure of the fetal immune system to molecular-level disturbances associated with infection may adversely affect fetal development and result in major health problems later in life. In this context, it may be necessary to rethink immunization of women during pregnancy [49].

## 4. Conclusions

This paper further develops the CDR's notion that adaptive arm of immune responses is activated primarily to regulate innate reactions and maintain integrity of a particular living system. It proposes that not only the already recognized adaptive repertoire selection and maintenance but also its function are all self-centered, i.e., dependent and focused on the state of self. Interpretation of regulatory processes and tolerance in allotransplantation suggests that adaptive effectors can exert their regulatory function only in a living system whose molecular patterns of self and its MHC “code” have originally served as a template for development and maintenance of that particular system's adaptive repertoire/specificities. Therefore, congruent/matching MHC-coded communications between innate and adaptive effectors provide regulation of immune responses, which are focused on that particular living system. Consistent with such a view of MHC-restricted adaptive immunity, immunosuppression-free operational tolerance in allotransplantation is more likely in patients with stable mixed chimerism, where both donor's and recipient's adaptive effectors maintain integrity of their respective MHC-matching tissues. The coexistence of MHC-disparate entities in such a living system also confirms that immunity is about adaptation and not elimination of a foreign: immunity is focused primarily on finding a sustainable equilibrium in a system to maintain its integrity, even in an allogeneic setting.

Pregnancy is an example of coexistence of MHC-disparate entities solely due to innate-level adaptation/molecular-level resettings to foreign molecular patterns. Adaptive immunity does not regulate those interactions: semiallogeneic or entirely allogeneic living systems during pregnancy are in close contact, but innate mechanisms suffice to maintain equilibrium states of involved living systems. The fact that in pregnancy cells are interchanged between MHC-disparate living systems and are detectable in mothers and their children long after birth further supports the notion that immunity is a matter of adaptation.
